# How Upward Moral Comparison Influences Prosocial Behavioral Intention: Examining the Mediating Role of Guilt and the Moderating Role of Moral Identity

**DOI:** 10.3389/fpsyg.2017.01554

**Published:** 2017-09-12

**Authors:** Heyun Zhang, Sisi Chen, Rong Wang, Jiang Jiang, Yan Xu, Huanhuan Zhao

**Affiliations:** ^1^Beijing Key Laboratory of Applied Experimental Psychology, National Demonstration Center for Experimental Psychology Education (Beijing Normal University), Faculty of Psychology, Beijing Normal University Beijing, China; ^2^School of Social Administration, Shanghai University of Political Science and Law Shanghai, China; ^3^College of Management, Shenzhen University Shenzhen, China; ^4^Department of Psychology, Shanghai Normal University Shanghai, China

**Keywords:** upward moral comparison, moral identity, guilt, prosocial behavioral intention, moderated mediation effect

## Abstract

Although it has been shown that exceptional good deeds exert influences on people's prosocial behavior and intention, we have known little about how common moral actions in our daily life. The present study aimed to examine how upward moral comparison influenced prosocial behavioral intention as well as to explore the mediating role of guilt and the moderating role of moral identity in the focal relationship. An experimental study was conducted with 162 Chinese undergraduates (103 women, 59 men) randomly assigned to an upward moral comparison condition, an upward competence comparison condition or a control condition. Results indicated that participants in the upward moral comparison condition experienced higher levels of guilt and exhibited stronger motivation to act prosocially, relative to participants in the other two conditions. That is to say, upward moral comparison induces guilty experience, and then increases people's prosocial behavioral intention. Moreover, we have found that moral identity internalization moderates the upward moral comparison-guilt relationship, and the indirect effect of upward moral comparison on prosocial behavioral intention via guilt. The implications of these findings were discussed.

Not all of us can do great things, but we can do small things with great love.

Mother Teresa

## Introduction

As a model of altruism, Mother Teresa's moral actions activate observers' moral emotions such as moral elevation. Moral elevation refers to a distinctive feeling of warmth and expansion that is accompanied by admiration, affection, and love for people with exemplary behaviors (Haidt, 2003). Moral elevation motivates observers to learn from the role models and demonstrate their own prosocial behaviors (Aquino et al., 2011). Not everyone can be Mother Teresa. However, the theory of social comparison suggests that others' behavior exerts a strong impact on people's self- perception and performance (Festinger, 1954). Therefore, focusing on moral actions in our daily life, the current study explored the influence of upward moral comparison on prosocial behavioral intention. Moreover, a moderated-mediating model was built to examine the mediating role of guilt and the moderating role of moral identity in the focal relationship.

### Upward moral comparison and prosocial behavioral intention

The theory of social comparison has posited that individuals acquire accurate self-appraisal through comparison with others (Festinger, 1954). There are two subtypes of social comparison: downward comparison and upward comparison. As a spontaneous human activity, upward and downward social comparisons occur frequently everyday, which often informs, enhances, and motivates people (Fiske, 2010). Downward social comparison enhances individuals' self-esteem by comparing with others who are worse off (Wills, 1981), whereas upward social comparison activates individuals' self-improvement motivation by comparing with others who are better off (Major et al., 1991; Lockwood and Kunda, 1997). Thus, relative to downward social comparison, upward social comparison is more likely to influence future behavior.

Upward moral comparison is a form of social comparison, whereby people compare themselves with others who are considered to be better in the moral domain (Monin, 2007). Prosocial behavior, as a form of moral behaviors (Baron, 1997; Batson et al., 2002), represents acts undertaken to protect or enhance the welfare of others (Schwartz and Bilsky, 1990), such as volunteer work (Schwartz and Fleishman, 1982), donating money (Frey and Meier, 2004), or blood (Zuckerman and Reis, 1978), and helping others who are in emergency situations (Schwartz and David, 1976). In this study, prosocial behavioral intention simply means that individuals have intention to do prosocial behaviors.

Upward moral comparisons can motivate people to learn from moral better ones and to engage in future moral behaviors by themselves. Monin (2007) has indicated that observing others' moral behaviors can trigger individuals' motivations to compare upward, resulting in the conclusion that their own morality is lacking. Then, the lack of morality motivates them to engage in prosocial behavior in order to restore their moral self-conception. Numerous studies have revealed that when individuals observe and recall others' moral actions, their moral behavioral intentions as well as actual moral behaviors also increase in the future (Cialdini et al., 1990; Goldstein et al., 2008; Aquino et al., 2011; Jordan et al., 2011; Dessi and Monin, 2012). Specifically, others' moral deeds could increase the motivation to help others (Bryan and Test, 1967; Rushton and Campbell, 1977). Some researches (Freeman et al., 2009; Aquino et al., 2011) have reported that an example of uncommon moral goodness can induce people's prosocial behavioral intention and actual helping behaviors (e.g., donating more money to a local charity). Moreover, some studies have shown that individuals' cooperation behaviors increase after observing their peers' good deeds (Dessi and Monin, 2012).

With above in mind, *we expected that upward moral comparison would exert positive impact on prosocial behavioral intention (Hypothesis 1)*.

### The mediating role of guilt

It is believed that upward moral comparison influences prosocial behavioral intention indirectly via moral emotions such as guilt. Monin (2007) has reported that people tend to experience assimilative emotions (e.g., elevation, inspiration, and admiration) when superior individuals are dissimilar to them. However, they will experience contrastive emotions (e.g., resentment, depression, and guilt) after comparing with superior individuals who are similar to them. The current study focused on moral actions in daily life, with peers or similar ordinary people as comparators, and we expected individuals to experience contrastive emotions when making upward social comparisons. Guilt, a kind of contrastive emotions, is a type of morally relevant and negatively valenced “self-conscious” emotion. It develops from awareness of failure to live up to an important self-imposed behavioral standard reflecting what is deemed good, correct, appropriate, or desirable (Haidt, 2003; Tangney et al., 2007; Ongley et al., 2014).

Extant studies have shown that individuals experience guilt when they are reminded of their previous immoral deeds (Bastian et al., 2011). In this study, we expect people will also display a tendency to experience guilt after making upward moral comparisons, for two possible reasons. First, although the failure to engage in moral behavior is not synonymous with engaging in immoral behavior (Schwartz and Bilsky, 1990; Jordan et al., 2011), people are likely to experience guilt when they feel responsible for the failure to live up to their standards or social norms (Malti et al., 2014). Second, guilt is an adaptive emotion, which can benefit individuals and their relationships in various ways (Baumeister et al., 1994; Tangney, 1995). In summary, there are two possible reasons why individuals might experience guilt when they compare themselves to better others. The first is that individuals feel that they have not lived up to their own standards or social norms, and the second is that guilt is an adaptive emotion, which can benefit individuals and their relationships. Based on above, *we expected that upward moral comparison could increase individuals' guilt (Hypothesis 2a)*.

Although guilt is a negative emotion, it can result in positive consequences. According to the self-completion theory, people eliminate guilt, and maintain a moral self-image by engaging in various types of prosocial behaviors (Tangney et al., 2007; Jordan et al., 2011; Xu et al., 2011), including helping strangers (Carlsmit and Gross, 1969; Konecni, 1972), volunteering (Quiles and Bybee, 1997; Zhong and Liljenquist, 2006), protecting the environment (Rees et al., 2015), reducing moral hypocrisy (Polman and Ruttan, 2012), and cooperating in social bargaining games (de Hooge et al., 2007). Therefore, *we hypothesized that guilt would increase individuals' prosocial behavioral intention (Hypozthesis 2b)*.

In addition to Hypotheses 2a and 2b, *we further expected guilt to play a mediating role in the relationship between upward moral comparison and subsequent prosocial behavioral intention (Hypothesis 3)*.

### The moderating role of moral identity

Although upward moral comparison is likely to affect prosocial behavioral intention via guilt, not all individuals who do not offer help homogeneously will experience high levels of guilt and have prosocial behavioral intentions. To some extent, heterogeneity of outcomes originate from individual characteristics, for example, moral identity, that moderate (i.e., buffer or exacerbate) the effect of upward moral comparison on guilt as well as prosocial behavioral intention. Aquino and Reed (2002) have proposed a trait-based conceptualization of moral identity, referring to “the degree to which being a moral person is important to an individual's identity” (Aquino and Reed, 2002; Narvaez et al., 2006). According to Aquino and Reed (2002) social-cognitive model of moral identity, a strong moral identity enhances the accessibility of knowledge structures and schemata that guide self-regulation and foster moral action (Hertz and Krettenauer, 2016). Based on this view, moral identity can render mechanisms of guilt more effective (Aquino et al., 2007). That is, individuals with high moral identity are more likely to feel a stronger moral obligation to show concern for the needs and interests of out-groups than those with low moral identity (Aquino et al., 2007; Winterich et al., 2012). Therefore, it is expected that the indirect relations between upward moral comparison and prosocial behavioral intention via guilt will be stronger for individuals with higher levels of moral identity.

Furthermore, moral identity consists of two dimensions: internalization and symbolization (Aquino and Reed, 2002). Internalization reflects the degree to which a set of moral traits is central to self-concept, while symbolization reflects the degree to which these traits are expressed publicly via the individual's actions in the world. To our knowledge, few previous studies have examined the reason why upward moral comparison evokes guilt. We infer that there are two potential reasons for the evocation of guilt in upward moral comparison: on one hand, people with high levels of internalization moral identity are likely to fail to achieve moral standards or social norms after upward moral comparisons; on the other hand, if people hold high levels of moral identity symbolization, they tend to express guilt to save their public self-image and to enhance their social relationships. If the former interpretation is right, moral identity internalization should moderate the relationship between upward moral comparison and guilt. Specifically, individuals with higher levels of moral identity internalization are more likely to interpret upward moral comparison situations as a failure to live up to their moral standards or social norms, resulting in guilt. However, if the latter interpretation is supported, moral identity symbolization should moderate such relationship. Specifically, individuals with higher levels of moral identity symbolization are more likely to express guilt to save their public self-image and enhance their social relationships.

In summary, *we hypothesized that individuals' moral identity (internalization, symbolization, or both) would play a moderating role in the relationship between upward moral comparison and guilt (Hypothesis 4)*.

**Figure 1:**
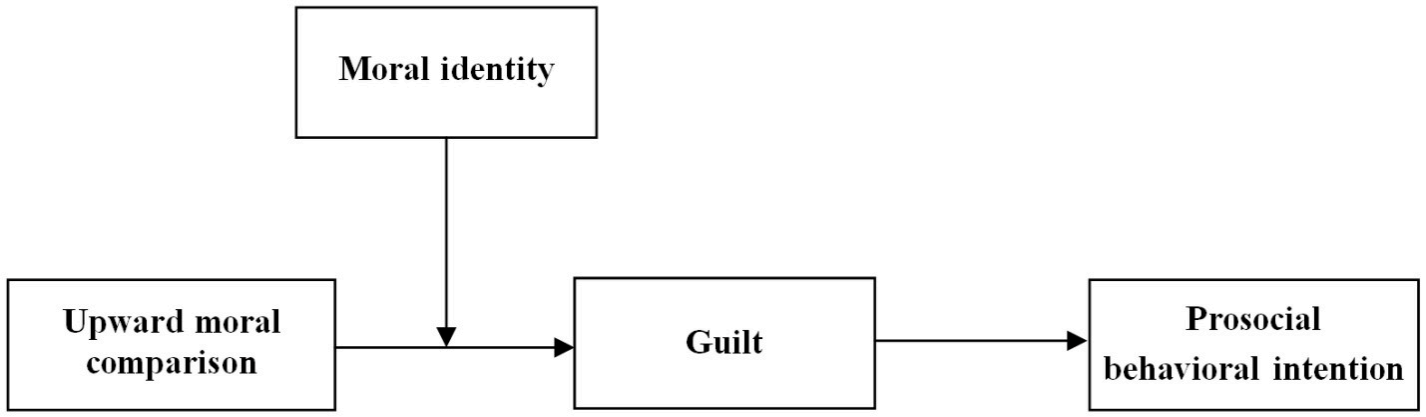
Conceptual framework for the study.

Based on hypotheses 3 and 4, moral identity was also expected to moderate the mediating effect of guilt on the relationship between upward moral comparison and prosocial behavioral intention. Specifically, the indirect effect of upward moral comparison on prosocial behavioral intention via guilt should be stronger (vs. weaker) for people with higher (vs. lower) levels of moral identity. *Such effect pattern is called moderated mediation* (Muller et al., 2005; Preacher et al., 2007). Figure 1 depicts the research model examined in the current study.

## Methods

### Participants

A total of 170 Chinese undergraduate students (109 women, 61 men) participated in the study voluntarily for the extra course credit. Their average age was 19.85 years (*SD* = 1.18, range: 18–25 years). All participants were randomly assigned into one of the following three groups: the upward moral comparison group (*n* = 61), upward competence comparison group *(n* = 56), and control group (*n* = 53).

### Procedure

At the beginning, participants were required to complete the Moral Identity Scale and to provide their demographic information. Then, they were randomly assigned into one of the three conditions. As shown in the following, we asked participants in each condition to recall a corresponding situation and to write an essay (of at least 100 words) to describe it.

#### Upward moral comparison condition

*Have you experienced a situation in which other people were more moral than you were in your daily life? An example of this type of situation follows: After a hard day's work, you are lucky enough to find a seat on a bus and sit down. During this time, an old man boards the bus. When you hesitate to give up your seat to the old man, another man stands up and gives his seat to the old man. You feel that the person is more moral than you are. Please use no fewer than 100 words to describe the events that you experienced*.

#### Upward competence comparison condition

*Have you experienced a situation in which other people were more competent than you were in your daily life? An example of this type of situation follows: In an examination, your friend answers the questions in a very difficult exam correctly, while you do not. You feel that your friend is more competent than you are. Please use no fewer than 100 words to describe the events that you experienced*.

#### Control condition

*Have you experienced something that impressed you during the last 2 weeks? Please use no fewer than 100 words to describe this*.

After writing the essay, all the participants were asked to answer one manipulation check question. Finally, we assessed participants' levels of guilt and prosocial behavioral intention.

### Measures

#### Moral identity

(Aquino and Reed, 2002) 10-item scale was used to assess the importance of moral identity to the self. The instrument is based on a conceptualization of moral identity as a schema organized around a set of moral trait associations (e.g., generosity, fairness, and compassion) and loads consistently on two dimensions: internalization and symbolization. Responses to the items are provided based on a 7-point Likert-type scale (1 = “strongly disagree,” 7 = “strongly agree”). Cronbach's α was 0.79 in the current study.

#### Guilt

Guilt was measured by using five-item guilt subscale from the State Shame and Guilt Scale (Marschall et al., 1994). A sample item is “I feel remorse and regret.” Responses for the guilt subscale items were provided based on a 7-point Likert scale (1 = “strongly disagree,” 7 = “strongly agree”). The item scores were averaged to provide a single score, and higher scores represent a stronger sense of guilt. Cronbach's α for the five guilt items was 0.79.

#### Prosocial intention

Prosocial behavioral intention was measured using five short scenarios (two about donating money to someone in need; two about donating time to be a volunteer to accompany the deaf-mute children and help your alumni; and one about donating blood to someone in need) (Please see the Supplement Material). One of the scenarios was as follows: “*A student in your school has a sudden, serious illness (leukemia), and his (her) classmates launch a fund raising activity for him (her). You have 100 yuan to spare; are you willing to donate the money to the student?*” Respondents were asked to rate their prosocial behavioral intention based on a 7-point Likert-type scale (from 1 = “very strongly unwilling” to 7 = “very strongly willing”). The prosocial behavioral intention index was calculated using the average score for the five scenarios. Higher values indicate greater willingness to engage in prosocial behaviors. In this research, its Cronbach's α was 0.61.

## Results^1^

### Manipulation check

In order to make sure that participants have made upward moral/competence comparisons with others, participants in the two experimental conditions were required to answer the following question: “Do you think that the person in the situation you described is more moral/competent than you?” Moreover, to make sure that participants in the control condition have involved in the experiment, they need to answer the following question: “Do you think that the story you wrote down is impressive?” We also ask two experiment assistants (Ph.D. students) to evaluate the contents of participants' essay.

According to the participants' answers and two experiment assistants' evaluations, eight participants' answers (Four in the experimental groups and four in the control group) were deleted as invalid data prior to testing the hypotheses. Analyses were performed based on valid data from 162 participants (Their average age was 19.84 years, *SD* = 1.16). Sample distributions are as follows: upward moral comparison (*n* = 59), upward competence comparison (*n* = 54) and control (*n* = 49) groups.

### Descriptive analyses

Means, standard deviations, and correlation coefficients for the key variables are presented in Table 1. Upward moral comparison was positively associated with guilt (*r* = 0.52, *p* < 0.001, 95% confidence interval [CI] = 0.39–0.63) and prosocial behavioral intention (*r* = 0.22, *p* < 0.01, 95% CI = 0.076–0.35). Prosocial behavioral intention was positively associated with both internalization (*r* = 0.36, *p* < 0.001, 95% CI = 0.20–0.53) and symbolization (*r* = 0.31, *p* < 0.001, 95% CI = 0.17–0.45). However, upward competence comparison was significantly negatively associated with guilt (*r* = −0.29, *p* < 0.001, 95% CI = −0.42 to −0.16).

**Table 1:** Descriptive statistics and correlations between key variables (*N* = 162).

**Variables**	***M***	***SD***	**1**	**2**	**3**	**4**	**5**	**6**	**7**
1. Moral comparison	–	–	1						
2. Competence comparison	–	–	−0.54^***^	1					
3. Guilt	3.46	1.31	0.52^***^	−0.29^***^	1				
4. Internalization	6.32	0.75	0.09	−0.05	0.06	1			
5. Symbolization	4.55	1.09	0.04	−0.13	0.07	0.30^***^	1		
6. Moral identity	5.43	0.74	0.07	−0.12	0.08	0.72^***^	0.88^***^	1	
7. Prosocial intention	5.42	0.91	0.22^**^	−0.13	0.28^***^	0.36^***^	0.31^***^	0.41^***^	1

*M, mean; SD, standard deviation. Moral comparison was dummy coded as follows: 1, upward moral comparison; 0, upward competence comparison; and 0, control condition. Competence comparison was dummy coded as follows: 0, upward moral comparison; 1, upward competence comparison; and 0, control condition*.

** p < 0.01;

*** *p < 0.001*.

### Between-group differences in guilt, moral identity, and prosocial behavioral intention

Three sets of one-way ANOVA analyses were performed to examine the influence of upward moral comparison on participants' guilt, moral identity, and prosocial behavioral intention (see Table 2). Moral identity did not differ significantly across groups, *F*_(2, 159)_ = 1.22, *p* > 0.05, Partial η^2^ = 0.02. However, results showed that guilt, *F*_(2, 159)_ = 29.51, *p* < 0.001, Partial η^2^ = 0.27, and prosocial behavioral intention, *F*_(2, 159)_ = 4.22, *p* < 0.05, Partial η^2^ = 0.05, differed significantly across the three groups. Moreover, results of LSD contrasts revealed that participants in the upward moral comparison group exhibited higher levels of guilt (*M* = 4.36) relative to those in the other two groups (upward competence comparison group: *M* = 2.91, *p* < 0.001, 95% CI = 1.02–1.86; control group: *M* = 2.98, *p* < 0.001, 95% CI = 95–1.81). Similarly, participants in the upward moral comparison group reported higher levels of prosocial behavioral intention (*M* = 5.69) relative to participants in the upward competence comparison (*M* = 5.26, *p* < 0.05, 95% CI = 0.099–0.77) and control (*M* = 5.28, *p* < 0.05, 95% CI = 0.075–0.76) groups. However, guilt (*p* = 0.79, 95% CI = −0.50–0.38) and prosocial behavioral intention (*p* = 0.93, 95% CI = −0.36–0.33) did not differ significantly between the upward competence comparison and control groups.

**Table 2:** One-way ANOVA comparing the three conditions (*N* = 162).

**Variables**	**Moral comparison (*****n*** = **59)**		**Competence comparison (*****n*** = **54)**		**Control condition (*****n*** = **49)**		***F***	**Partial η^2^**
	***M***	***SD***	***M***	***SD***	***M***	***SD***		
Guilt	4.36	1.16	2.91	0.97	2.98	1.25	29.51^***^	0.27
Internalization	6.40	0.56	6.26	0.98	6.27	0.65	0.59	0.01
Symbolization	4.60	0.94	4.35	1.19	4.72	1.12	1.60	0.02
Moral identity	5.50	0.61	5.31	0.85	5.49	0.76	1.22	0.02
Prosocial intention	5.69	0.84	5.26	0.97	5.28	0.88	4.22^*^	0.05

*M, mean; SD, standard deviation*.

* p < 0.05;

*** *p < 0.001*.

### The role of guilt: mediation analysis

As moral identity, guilt, and prosocial behavioral intention did not differ significantly between the upward competence comparison and control groups, both groups were coded as 0, while the upward moral comparison group was coded as 1. Based on Baron and Kenny (1986) guidelines for mediation analysis, several multiple linear regression analyses were performed to examine the mediating role of guilt in the relationship between upward moral comparison and prosocial behavioral intention.

After controlling gender and age, guilt was found to mediate the association between upward moral comparison and prosocial behavioral intention, with the following patterns: (1) Upward moral comparison was positively associated with guilt (see Equation 1 of Table 3; *B* = 1.36, *p* < 0.001, 95% CI = 0.97–1.75) and prosocial behavioral intention (see Equation 2 of Table 3; *B* = 0.40, *p* < 0.05, 95% CI = 0.09–0.71); (2) guilt was significantly associated with prosocial behavioral intention (see Equation 3 in Table 3; *B* = 0.15, *p* < 0.05, 95% CI = 0.03–0.28), and the coefficient for the upward moral comparison group was no longer significant (*B* = 0.19, *p* > 0.05, 95% CI = −0.15–0.54). Above results supported that guilt fully mediated the relationship between upward moral comparison and prosocial behavioral intention (Baron and Kenny, 1986). Results of the Sobel test also revealed that upward moral comparison exerted a significant indirect effect on prosocial behavioral intention via guilt (Sobel = 2.33, *p* < 0.05), providing support for Hypothesis 3.

**Table 3:** Results of the regression analysis of mediation (*N* = 162).

**Predictor**	**Equation 1**			**Equation 2**			**Equation 3**		
	**(criterion: guilt)**			**(criterion: prosocial intention)**			**(criterion: prosocial intention)**		
	***B***	***SE***	**95% CI**	***B***	***SE***	**95% CI**	***B***	***SE***	**95% CI**
Constant	3.83^*^	1.64	0.59, 7.07	5.39^***^	1.30	2.81, 7.96	4.80^***^	1.31	2.22, 7.38
Gender	−0.16	0.19	−0.53, 0.21	−0.17	0.15	−0.46, 0.13	−0.14	0.15	−0.43, 0.15
Age	−0.04	0.08	−0.20, 0.12	−0.002	0.06	−0.13, 0.12	0.004	0.06	−0.12, 0.13
UMC	1.36^***^	0.20	0.97, 1.75	0.40^*^	0.16	0.09, 0.71	0.19	0.18	−0.15, 0.54
Guilt							0.15^*^	0.06	0.03, 0.28
Δ*R*^2^								0.035^*^	
Model *R*^2^		0.27^***^			0.058^*^			0.93^**^	
*F*		19.95			3.24			4.03	

*CI, confidence interval; SE, standard error; UMC, upward moral comparison. Each set of columns shows the regression equation for the criterion in the column heading. UMC was dummy coded as follows: 1, 0, 0; Gender was dummy coded as follows: 0, female and 1, male*.

* p < 0.05;

** p < 0.01;

*** *p < 0.001*.

### The roles of moral identity and guilt: moderated mediation analysis

In terms of the moderated mediation model (Hypothesis 4), the fulfillment of several conditions was required to demonstrate a first-stage moderated mediation model (Muller et al., 2005; Edwards and Lambert, 2007; Preacher et al., 2007; Hayes, 2015): (1) a significant effect of upward moral comparison on prosocial behavioral intention; (2) a significant effect of the interaction between upward moral comparison and moral identity (internalization, symbolization, or both) in predicting guilt; (3) a significant effect of guilt on prosocial behavioral intention; and (4) a significant difference in the indirect effects of upward moral comparison on prosocial behavioral intention between high and low levels of moral identity people.

**Figure 2:**
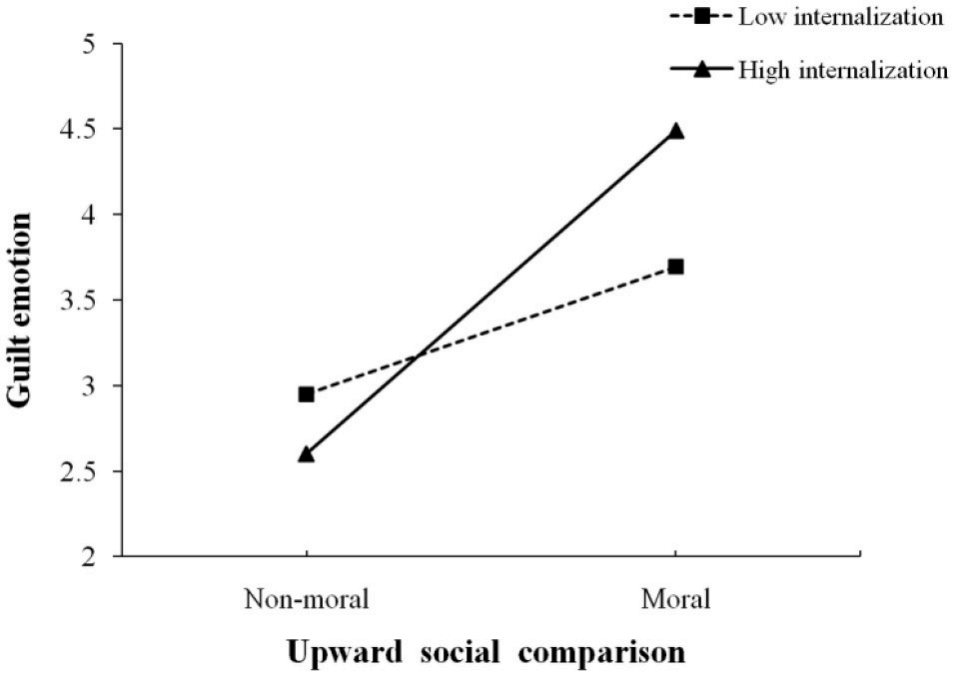
Simple slopes for the effect of the interaction between upward moral comparison and moral identity internalization on guilt.

We conducted a series of regression analyses to test the first three conditions. In Model 1, guilt was regressed on the control variables (gender and age), upward moral comparison, moral identity internalization and symbolization, and the interaction terms of upward moral comparison and moral identity (internalization and symbolization). In Model 2, prosocial behavioral intention was regressed on the control variables (gender and age), upward moral comparison, moral identity (internalization and symbolization), and the interaction terms of upward moral comparison and moral identity (internalization and symbolization). In Model 3, prosocial behavioral intention was regressed on the control variables (gender and age), upward moral comparison, moral identity (internalization and symbolization), guilt, and the interaction terms of upward moral comparison and moral identity (internalization and symbolization). Results were as follows: (1) upward moral comparison was positively associated with prosocial behavioral intention (see Model 2, β = 0.20, *p* < 0.01, 95% CI = 0.09–0.66), and (2) the upward moral comparison × internalization interaction accounted for an additional 4% of the variance in guilt (see Model 1, Δ*R*^2^ = 0.04, *p* < 0.05). We then conducted a simple-slope test (Aiken and West, 1991; Preacher et al., 2006) to interpret the pattern of such interaction (see Figure 2). The simple slope of the regression of upward moral comparison on guilt was significant when moral identity internalization was high (i.e., 1 *SD* above the mean; β = 0.70, *p* < 0.001, 95% CI = 0.65–1.19). The relationship between upward moral comparison and guilt was also significant when moral identity internalization was low (i.e., 1 *SD* below the mean; β = 0.27, *p* < 0.05, 95% CI = 0.07–0.64); however, the simple slope was decreased from 0.70–0.27. (3) In Model 3, results showed that guilt was positively related to prosocial behavioral intention (β = 0.21, *p* < 0.05, 95% CI = 0.03–0.27).

To test the fourth condition, we performed regression analysis according to the specifications established by Andrew Hayes' PROCESS macros for SPSS (Hayes, 2013) to examine the moderated mediation model. Results showed that the index value for moderated mediation was significant [index = 0.12, *SE*(Boot) = 0.06, BootLLCI = 0.03, BootULCI = 0.26]. Then, we used the bootstrap method (2000 bootstrap samples) to compare the indirect effect of upward moral comparison on prosocial behavioral intention via guilt across different levels of moral identity internalization (Edwards and Lambert, 2007; Preacher et al., 2007). Results showed that the indirect effects differed significantly between individuals with high (indirect effect = 0.30, 95% CI = 0.07–0.59) and low moral identity internalization (indirect effect = 0.12, 95% CI = 0.01–0.33), and the difference was significant (difference = 0.18, 95% CI = 0.04–0.44). Therefore, the fourth condition was fulfilled.

## Discussion

An old Chinese saying tell us that: Never fail to do anything good that you deem petty; never dare to do anything evil that you consider trivial. That is to say, individuals feel that they have an obligation to engage in good deeds, even those are considered to be small acts of goodness. Previous research has shown that when people are exposed to extraordinary moral goodness, they experience moral elevation, which increases their motivations to engage in prosocial behaviors (Aquino et al., 2011). However, the extraordinary good deeds do not always occur in the presence of others; thus, people may feel that such extraordinary good deeds are irrelevant to them. Conversely, people observe small good deeds easily. In the current study, by focusing on events happened in our daily life, we explored the relationship between upward moral/competence comparisons and prosocial behavioral intention, as well as the underlying psychological mechanisms of such relationship. The main findings are summarized and discussed below.

Our results showed that when people were confronted with daily moral goodness performed by others, their prosocial behavioral intention increased greatly. Moreover, correlation analysis revealed that upward moral comparison rather than competence comparison was positively associated with prosocial behavioral intention. In our study, participants in the upward moral comparison group reported stronger intention to engage in prosocial behaviors relative to those in the other two groups. These findings are consistent with those of previous studies, which indicated that moral action in others increased individuals' prosocial behavioral intention (Bryan and Test, 1967; Zhong and Liljenquist, 2006; Aquino et al., 2011).

In line with our hypotheses, we also found that guilt played a mediating role in the relationship between upward moral comparison and prosocial behavioral intention. Such finding extends those of previous studies, most of which examined the effects of others' extraordinary behavior on participants' feelings and subsequent behaviors (Algoe and Haidt, 2009; Schnall et al., 2010; Aquino et al., 2011). Previous researches have suggested that uncommon moral good deeds can induce moral elevation, which will increase prosocial behavioral intentions and actual prosocial behaviors. However, in the present study, we found that even when individuals compared themselves with their peers or other ordinary people around them who engaged in common good deeds, they experienced guilt for failing to provide help to people in need. According to the social comparison theory (Festinger, 1954; Buunk, 1995; Monin, 2007), upward moral comparison can threaten individuals' self-image and cause them to feel that they have failed to live up to their moral standards. Many theories have posited that peoples' guilt stems from the awareness of their failure to live up to important standards (Haidt, 2003; Tangney et al., 2007). Moreover, the self-completion theory suggests that guilt, as a form of tension resulting from previous behaviors (e.g., failing to offer help to those in need), is likely to motivate people to demonstrate moral behaviors (e.g., donating or volunteering) (Gollwitzer, 1986; Tangney et al., 2007; Jordan et al., 2011; Xu et al., 2011, 2012). Therefore, when comparing with others who have performed common good deeds, people have a tendency to experience guilt, and then increase their prosocial behavioral intention.

The obtained findings also revealed that moral identity internalization rather than moral identity symbolization moderated the relationship between upward moral comparison and guilt. Our moderated mediation model was also supported. Specifically, when comparing with others in the moral domain, people with high levels of internalization moral identity were more likely to experience guilt and engage in prosocial behavior relative to those with low levels of internalization moral identity. Similar to our study, Shao et al. (2008) also suggested that moral identity influenced peoples' interpretation of and response to situations involving moral judgment and choice. In the current study, the interaction between upward moral comparison and moral identity predicted guilt significantly, which increased prosocial behavioral intention (see Table 4). However, we found that only the effect of interaction between moral identity internalization and upward moral comparison on guilt was significant. Schwartz (1977) has proposed the Norm Activation Model (NAM), which has been successfully applied in predicting a diversity of prosocial intentions and behaviors (Schwartz, 1977). As expected on the basis of the NAM, a strong moral obligation to act prosocially is associated with higher levels of prosocial behavioral intentions. Aquino and Reed (2002) have posited that moral identity internalization reflects the degree to which a set of moral traits is central to one's self-concept, and moral identity symbolization reflects the degree to which these traits are expressed publicly through the people's actions. The moral identity internalization is a kind of personal norms (PN), referred to as feeling a “moral obligation to perform specific actions” (Schwartz and Howard, 1981). Therefore, it is believed that participants in the upward moral comparison group experienced guilt due to their belief that they had failed to live up to their standards.

**Table 4:** Results of hierarchical regression analysis for moderated mediation (*N* = 162).

**Predictor**	**Model 1**			**Model 2**			**Model 3**		
	**Guilt**			**Prosocial intention**			**Prosocial intention**		
	***B***	***SE***	**95% CI**	***B***	***SE***	**95% CI**	***B***	***SE***	**95% CI**
Constant	8.09^***^	1.73	4.66, 11.51	6.65^***^	1.23	4.22, 9.08	6.65^***^	1.23	4.22, 9.08
Gender	−0.34	0.21	−0.75, 0.08	−0.22	0.15	−0.51, 0.07	−0.22	0.15	−0.51, 0.07
Age	−0.23^**^	0.09	−0.40, −0.06	−0.06	0.06	−0.18, 0.06	−0.06	0.06	−0.18, 0.06
	*R*^2^ = 0.05^*^Δ*R*^2^ = 0.05^*^			*R*^2^ = 0.02 Δ*R*^2^ = 0.02			*R*^2^ = 0.02 Δ*R*^2^ = 0.02		
	Δ*F =* 4.58^*^			Δ*F* = 1.48			Δ*F* = 1.48		
UMC	1.36^***^	0.20	0.97, 1.75	0.38^**^	0.14	0.09, 0.66	0.17	0.16	−0.15, 0.49
MI_I	−0.02	0.13	−0.27, 0.23	0.34^***^	0.09	0.16, 0.52	0.34^***^	0.10	0.14, 0.53
MI_S	0.05	0.09	−0.12, 0.22	0.18^**^	0.06	0.06, 0.31	0.17^**^	0.06	0.04, 0.30
	*R*^2^ = 0.28^***^Δ*R*^2^ = 0.22^***^			*R*^2^ = 0.21^***^Δ*R*^2^ = 0.20^***^			–		
	Δ*F* = 15.96^***^			Δ*F* = 12.93^***^					
MI_I × UMC	0.67^*^	0.31	0.07, 1.28	0.09	0.23	−0.36, 0.54	−0.01	0.23	−0.46, 0.44
MI_S × UMC	0.21	0.19	−0.17, 0.60	−0.05	0.14	−0.33, 0.24	−0.08	0.14	−0.36, 0.20
	*R*^2^ = 0.31^***^Δ*R*^2^ = 0.04^*^			*R*^2^ = 0.21^***^Δ*R*^2^ = 0.001			—		
	Δ*F =* 4.15^*^			Δ*F* = 0.10					
Guilt	—	—	—	—	—	—	0.15^*^	0.06	0.03, 0.27
							*R*^2^ = 0.25^***^Δ*R*^2^ = 0.23^***^		
							Δ*F* = 7.72^***^		

*MI_I, moral identity internalization; MI_S, moral identity symbolization; UMC, upward moral comparison*.

* p < 0.05;

** p < 0.01;

*** *p < 0.001*.

### Implications

Our study made two major contributions to the current knowledge regarding the psychological mechanisms underlying the influence of common moral goodness on prosocial behavioral intention. First, previous research has examined the effect of uncommon moral goodness on people's feelings and subsequent behaviors (Algoe and Haidt, 2009; Schnall et al., 2010; Aquino et al., 2011). We explored the influence of daily life moral goodness on peoples' prosocial behavioral intention as well as examined the mediating role of guilt in such relationship. Second, Fiske (2010) has concluded that comparison is only natural, but the collateral damage reveals envy upward and scorn downward. However, the present study suggests the positive impacts of upward moral comparison. Our results revealed that only the interaction between moral identity internalization and upward moral comparison predicted participants' guilt, which increased their prosocial behavioral intention.

The current study also has some practical implications. In particular, the findings suggest that upward moral comparison in daily life increases our intention to engage in future moral behaviors. If we want someone act prosocially, one of the possible ways is encouraging people to compare with moral others (e.g., designing some banners with “others can do this, you also can” on it). Similarly put, the government also can make people compare with others by posting some slogans to induce citizens' prosocial intentions.

### Limitations and directions for future research

The study was subject to several limitations. First, it involved the assessment of prosocial intention rather than actual behavior. Previous studies have shown that there is discrepancy between judgment about prosocial behavior and actual behavior (Patil et al., 2017). Future research should extend our findings using behavioral measures such as actual donation activity. Second, our participants were all recruited from China, a country with a collectivist culture. People in collectivist societies are more concerned about social relationships relative to people in individualist societies (Markus and Kitayama, 1991). Future researchers are encouraged to replicate our study under different cultural contexts. We believe that cross-cultural examination of the relationship between and mechanisms underlying upward moral comparison and prosocial behavior would be an interesting and valuable direction for future research.

## Conclusions

The current study deepened our understanding regarding the impacts of social comparisons on our daily behaviors. Specifically, by employing an experiment, we found that upward moral comparison positively and indirectly influenced peoples' prosocial behavioral intention via guilt. Moreover, moral identity internalization strengthened the upward moral comparison-guilt relationship, as well as the aforementioned mediating effect of guilt. Therefore, as suggested by Mother Teresa, our society needs not only exceptional good deeds but also common moral goodness in daily life.

## Ethics statement

Prior to the research, ethical approval was obtained from the Committee of Protection of Subjects at Beijing Normal University. All participants were required to read and approve the informed consent before participating in this research.

## Author contributions

Conceived and designed the experiments: HeZ, SC, and JJ. Performed the experiments: SC and JJ. Analyzed the data: HeZ, RW, and JJ. Contributed to the writing of the manuscript: HeZ, SC, RW, JJ, YX, and HuZ.

### Conflict of interest statement

The authors declare that the research was conducted in the absence of any commercial or financial relationships that could be construed as a potential conflict of interest. The reviewer, NM, and handling Editor declared their shared affiliation.

## Footnotes

^1^The original data has been uploaded to the Supplement Material Section.
