# Corrigendum: How Upward Moral Comparison Influences Prosocial Behavioral Intention: Examining the Mediating Role of Guilt and the Moderating Role of Moral Identity

**DOI:** 10.3389/fpsyg.2017.01815

**Published:** 2017-10-17

**Authors:** Heyun Zhang, Sisi Chen, Rong Wang, Jiang Jiang, Yan Xu, Huanhuan Zhao

**Affiliations:** ^1^Beijing Key Laboratory of Applied Experimental Psychology, National Demonstration Center for Experimental Psychology Education, Faculty of Psychology, Beijing Normal University, Beijing, China; ^2^School of Social Administration, Shanghai University of Political Science and Law, Shanghai, China; ^3^College of Management, Shenzhen University, Shenzhen, China; ^4^Department of Psychology, Shanghai Normal University, Shanghai, China

**Keywords:** upward moral comparison, moral identity, guilt, prosocial behavioral intention, moderated mediation effect

In the original article, the authors neglected to include the supplementary material. The original article has now been updated and the original data and relevant materials have been added to the section of supplementary material.

The authors apologize for this error and state that this does not change the scientific conclusions of the article in any way.

## Conflict of interest statement

The authors declare that the research was conducted in the absence of any commercial or financial relationships that could be construed as a potential conflict of interest.

