# Supplementary material for: How Upward Moral Comparison Influences Prosocial Behavioral Intention: Examining the Mediating Role of Guilt and the Moderating Role of Moral Identity
Source: Front Psychol. 2017 Sep 12;8:1554. doi: 10.3389/fpsyg.2017.01554 (PMC5604077; doi:10.3389/fpsyg.2017.01554)
Supplement: Supplementary file 1 [file Presentation1.pdf]

Supplementary materials

Prosocial intention. Prosocial behavioral intention was measured using five short scenarios (two about donating money to someone in need; two about donating time to be a volunteer to accompany the deaf-mute children and help your alumni; and one about donating blood to someone in need). Respondents were asked to rate their prosocial behavioral intention based on a 7-point Likert-type scale (from 1 = "very strongly unwilling" to 7 = "very strongly willing").

The scenarios were as follows:

"A student in your school has a sudden, serious illness (leukemia), and his (her) classmates launch a fund raising activity for him (her). You have 100 yuan to spare; are you willing to donate the money to the student?"

very strongly unwilling 1……2……3……4……5……6……7 very strongly willing

"Haidian Disabled Persons' Federation needs to recruit a group of volunteers, who need accompany the deaf-mute children to learn two hours per week. You just have free time every week; are you willing to spend your time to accompany the deaf-mute children?"

very strongly unwilling 1……2……3……4……5……6……7 very strongly willing

"Your school will hold a school celebration for seven days and need some volunteers to guide the alumni to visit the campus. Assuming that you have free time in those 7 days; are you willing to participate?"

very strongly unwilling 1……2……3……4……5……6……7 very strongly willing

"Your school's foundation is raising money for children from poor mountainous areas. The money will be used to buy textbooks and writing materials for the children. You have 100 yuan to spare; are you willing to donate the money to the student?"

very strongly unwilling 1……2……3……4……5……6……7 very strongly willing

“There is a shortage of blood bank resource in Beijing and it is urgent for someone to donate blood. Assuming that your blood type would fit with the required type; are you willing to participate in blood donation?”

very strongly unwilling 1……2……3……4……5……6……7 very strongly willing
