# Supplementary material for: How Upward Moral Comparison Influences Prosocial Behavioral Intention: Examining the Mediating Role of Guilt and the Moderating Role of Moral Identity
Source: Front Psychol. 2017 Sep 12;8:1554. doi: 10.3389/fpsyg.2017.01554 (PMC5604077; doi:10.3389/fpsyg.2017.01554)
Supplement: Supplementary file 2 [file DataSheet1.zip › Prosocial intention materials.docx]

**Supplementary materials**

***Prosocial intention.*** Prosocial behavioral intention was measured using five short scenarios (two about donating money to someone in need; two about donating time to be a volunteer to accompany the deaf-mute children and help your alumni; and one about donating blood to someone in need). Respondents were asked to rate their prosocial behavioral intention based on a 7-point Likert-type scale (from 1 = “very strongly unwilling” to 7 = “very strongly willing”).

**The scenarios were as follows:**

*“A student in your school has a sudden, serious illness (leukemia), and his (her) classmates launch a fund raising activity for him (her). You have 100 yuan to spare; are you willing to donate the money to the student?”*

very strongly unwilling 1……2……3……4……5……6……7 very strongly willing

*“Haidian Disabled Persons’ Federation needs to recruit a group of volunteers, who need accompany the deaf-mute children to learn two hours per week. You just have free time every week; are you willing to spend your time to accompany the deaf-mute children?”*

very strongly unwilling 1……2……3……4……5……6……7 very strongly willing

*“Your school will hold a school celebration for seven days and need some volunteers to guide the alumni to visit the campus. Assuming that you have free time in those 7 days; are you willing to participate?”*

very strongly unwilling 1……2……3……4……5……6……7 very strongly willing

*“Your school’s foundation is raising money for children from poor mountainous areas. The money will be used to buy textbooks and* *writing materials for the children. You have 100 yuan to spare; are you willing to donate the money to the student?”*

very strongly unwilling 1……2……3……4……5……6……7 very strongly willing

*“There is a shortage of blood bank resource in Beijing and it is urgent for someone to donate blood. Assuming that your blood type would fit with the required type; are you willing to participate in blood donation?”*

very strongly unwilling 1……2……3……4……5……6……7 very strongly willing
